# Cytosolic glutamine synthetase is important for photosynthetic efficiency and water use efficiency in potato as revealed by high-throughput sequencing QTL analysis

**DOI:** 10.1007/s00122-015-2573-2

**Published:** 2015-07-12

**Authors:** Kacper Piotr Kaminski, Kirsten Kørup, Mathias Neumann Andersen, Mads Sønderkær, Mette Sondrup Andersen, Hanne Grethe Kirk, Kåre Lehmann Nielsen

**Affiliations:** Department of Chemistry and Bioscience, Aalborg University, Fredrik Bajers Vej 7H, 9220 Aalborg Øst, Denmark; Department of Agroecology, Faculty of Science and Technology, Aarhus University, Blichers Allé 20, 8830 Tjele, Denmark; Danish Potato Breeding Foundation, Grindstedvej 55, 7184 Vandel, Denmark; Department of Biotechnology, Chemistry and Environmental Engineering, Aalborg University, Fredrik Bajers Vej 7H, 9220 Aalborg Øst, Denmark

## Abstract

**Key message:**

**WUE phenotyping and subsequent QTL analysis revealed cytosolic GS genes importance for limiting N loss due to photorespiration under well-watered and well-fertilized conditions.**

**Abstract:**

Potato (*Solanum tuberosum* L.) closes its stomata at relatively low soil water deficits frequently encountered in normal field conditions resulting in unnecessary annual yield losses and extensive use of artificial irrigation. Therefore, unraveling the genetics underpinning variation in water use efficiency (WUE) of potato is important, but has been limited by technical difficulties in assessing the trait on individual plants and thus is poorly understood. In this study, a mapping population of potatoes has been robustly phenotyped, and considerable variation in WUE under well-watered conditions was observed. Two extreme WUE bulks of clones were identified and pools of genomic DNA from them as well as the parents were sequenced and mapped to reference potato genome. Following a novel data analysis approach, two highly resolved QTLs were found on chromosome 1 and 9. Interestingly, three genes encoding isoforms of cytosolic glutamine synthase were located in the QTL at chromosome 1 suggesting a major contribution of this enzyme to photosynthetic efficiency and thus WUE in potato. Indeed, Glutamine synthetase enzyme activity of leaf extracts was measured and found to be correlated with contrasting WUE phenotypes.

**Electronic supplementary material:**

The online version of this article (doi:10.1007/s00122-015-2573-2) contains supplementary material, which is available to authorized users.

## Introduction

Potato is the third most important food crop worldwide (FAOSTAT 2013). It has a higher WUE potential than cereals (Vreugdenhil et al. 2007), has a high harvest index, and is a very space efficient crop potentially producing roughly twice as many calories per hectare than cereals (FAOSTAT 2013); a desirable fact in a future scenario of limited agricultural land. However, potato is sensitive to even mild water stress. It closes its stomata at relatively low soil water deficits, which results in a considerable higher yield decrease compared to cereals (Porter et al. 1999). Potatoes are unique as a crop species in the sense that it is the only major crop, where a stolon-derived tuber serves as a sink organ. Therefore, it is not known how accurately information gained from studies on other crops (e.g., the closely related tomato) or model plants such as *Arabidopsis thaliana* can be transferred to potato.

Potato breeding was until recently almost exclusively conducted as classical selective breeding, where two parents deemed suitable are crossed to generate a number of offspring, which are subsequently selected based on the evaluation of a number of phenotypical trait scores (Gepts 2009). The number of traits scored in potato accumulates during the up to 10 years long evaluation period and can amount to as much as 75–100 traits scored in multiple years and locations (Collard and Mackill 2008). This process is both expensive and time consuming; particularly for potato because tubers have special storage requirements with regard to temperature and humidity. Compact storage and growth of potato are also limited by the size of tubers and plants. In the last few decades in many plants species, developments in molecular biological methods and the molecular genetics underlying important traits have paved the way for the use of molecular breeding methods such as marker-assisted selection (MAS) and genomic selection (Gao et al. 2012). MAS makes use of molecular markers for single dominant traits of fundamental importance, or of major contributing genes to polygenetic traits, which are either expensive to assess or which can only be assessed at a late stage after long and expensive growth and maintenance of many offspring. The relatively expensive necessary genotyping cost (although continuously decreasing) per clone is counterbalanced by the fact that it can be applied to young material and thus enable early selection of candidate breeding clones, thus limiting growth and storage costs. Following the elucidation of the potato genome sequence (Potato Genome Sequencing Consortium et al. 2011), the implementation of MAS or even more advanced genomics assisted methods of breeding is now possible. However, useful and comprehensive molecular markers linked to traits facilitating MAS are still lacking in potato. Also, identification of the causal genes underlying important traits is not very advanced, and thus, our understanding of the genetics and biochemistry contributing to and regulating agronomic important characteristics of this unique tuber crop is limited.

Water use efficiency (WUE) is a complicated trait to assess since phenotyping of WUE requires specialized facilities and is very laborious, particularly at leaf level where the use of sophisticated photosynthesis measuring systems is necessary (Chen et al. 2011). Hence, focused breeding for WUE has generally been underdeveloped across crop species (Chen et al. 2011). Here, we analyze a potato mapping population of 144 offspring originating from a reciprocal cross of two diploidal potato lines 90-HAF-01 (*S. tuberosum*_1_ × *S. tuberosum*_2_) (HAF) and 90-HAG-15 (*S. tuberosum*_3_ × *S. sparsipilum*) (HAG). The mapping population together with the parents was phenotyped twice in two consecutive years in over 50 combinations of environmental conditions, all well watered, in order to access WUE. A total of 14,000 photosynthetic measurements were performed. In an approach essentially similar to SHOREmap (Schneeberger et al. 2009), bulk segregant analysis was then conducted for clones of low- and high-WUE phenotypes. Genome re-sequencing of both parents and WUE bulks was performed, and the sequenced reads were mapped to draft potato genome sequence (Potato Genome Sequencing Consortium et al. 2011). De novo molecular markers in form of single nucleotide polymorphisms (SNPs) were developed based on parental mappings, and quantitative trait loci (QTLs) were subsequently detected by comparing the nucleotide distribution of the bulks and the parents, using a novel statistical method identifying regions of the genome that were likely to be non-randomly selected in the bulks and thus constitute QTLs for WUE.

## Materials and methods

### Plant cultivation and phenotyping

A population designated HCDHDN originating from two reciprocal crosses between 90-HAF-01 (*S. tuberosum*_1_ × *S. tuberosum*_2_) and 90-HAG-15 (*S. tuberosum*_3_ × *S. sparsipilum*) (Sørensen et al. 2008) was used in this study. For all experiments, plants were grown under controlled well-watered and well-fertilized conditions in climate chambers. In 2010, a set of measurements were performed using one replicate of 146 different genotypes including the parents. Potato tubers (35–55 mm) were pre-sprouted at 12 °C for 2 weeks and then planted at 5 cm depth in pots. Two sets of measurements of net photosynthesis and transpiration rates using the portable photosynthesis system (CIRAS-2, PP Systems, Amesbury, MA, US) were performed under various combinations of environmental conditions, with different values of air temperature (*T*), relative air humidity (*Rh*), CO_2_ concentration [CO_2_], and light. The potato tubers were planted on June 7th (1st set of measurements) and June 24th (2nd set of measurements). Measurements started 8 weeks after planting. The 1st set of measurements lasted for 2 weeks, from 2nd until 16th of August, whereas 2nd set lasted also 2 weeks, from 18th until 31st of August. In both sets, temperature during night was kept for 12 h at 12 °C with relative humidity of 85 % and light at 0 PAR. During day phenotyping, each combination of environmental conditions was kept constant for 8 h allowing for plant adaptation and reliable measurements of *pWUE*. The first set of measurements was conducted either under a fixed CO_2_ concentration of 380 ppm, while plants were exposed to combinations of three different temperature levels (12, 20, and 28 °*C*), *Rh* (40, 65, and 85 %), and light (200, 700, and 1200 µmol m^−2^ s^−1^), or under a fixed temperature level at 20 °C while exposed to combinations of [CO_2_]. Three levels of *Rh* (40, 65, and 85 %), light (200, 700, and 1200 µmol m^−2^ s^−1^), and [CO_2_] (380, 700, and 1000 ppm) were applied. The second set of measurements (data not shown) was performed either under [CO_2_] fixed at 380 ppm with alternating temperature (14, 21, and 28 °C), *Rh* (50, 80 %), and light (200, 700, and 1200 µmol m^−2^ s^−1^), or under a relatively high fixed [CO_2_] at 1000 ppm, while exposed to combinations of three temperature levels (14, 21, and 28 °C), *Rh* (50, 80 %), and light (200, 700, and 1200 µmol m^−2^ s^−1^). In all experiments, a total of 14,000 measurements were performed (8000 performed in 2010, based on which bulk segregation was performed). Following photosynthetic measurements, aboveground plant materials (leaves, stems, and flowers) as well as tubers were harvested, dried at 80 *°*C overnight, and weighed. In addition, the roots dry mass for each clone were determined following the second set of measurements. All plant pots including sphagnum, potato roots, and tubers were weighed before and after measurements. The amount of water used by each plant during the experiment was calculated. Irrigation *WUE* (*iWUE)* defined as dry matter (DM) per water use (WU) and photosynthetic WUE (*pWUE*) defined as ratio of net photosynthetic rate (*A*) to net transpiration rate (*E*) were calculated. Based on the distribution of WUE calculated using the first and second set of measurements, a low WUE bulk (21 clones) and a high WUE bulk (24 clones) were identified. The low WUE bulk consisted only of clones with either *pWUE* or *iWUE* lower than 3.50 (µmolCO_2_/mmolH_2_O and g/l, respectively), and the high WUE bulk was created and consisted of clones with either *pWUE* or *iWUE* higher than 4.75 (µmolCO_2_/mmolH_2_O and g/l, respectively). Among the 54 offspring used in the 2nd year of measurements, 11 clones belonged to the low WUE bulk and 13 clones to the high WUE bulk.

### DNA extraction and sequencing

Leaf samples were taken from each plant, frozen in liquid nitrogen, and stored at −80 °C until further processing. DNA extraction was performed separately for each plant by homogenizing 100–200 mg of leaf tissue by subjecting the sample to three cycles of 10 s (with 5 s pause in between) homogenization at 6500 rpm using a Precellys mechanical homogenizer (Bertin Technologies, France) in a Precellys CK14 tissue homogenization tube (including beads). Disrupted samples were then subjected to DNA extraction by DNeasy^®^ Plant Mini kit (QIAGEN^®^ group) following the manufacturer’s instructions. DNA purity was evaluated by 1 % TAE-agarose gel electrophoresis) and spectroscopic analysis by NanoDrop^®^ Spectrophotometer ND-1000 (Thermo Scientific, Wilmington, De, USA). Subsequently, PicoGreen^®^ dsDNA quantitation assay was performed for all DNA samples facilitating pooling of equal amounts of DNA from each sample for the low and high WUE bulks prior to processing with TruSeq™ sample preparation kit and paired-end HiSeq 2000 (Illumina, San Diego, USA) sequencing. Image analysis and base calling were performed using Hiseq control software version 1.5.15.1 and CASAVA version 1.8.2, respectively.

### Reference mapping and variant detection

Using the Genomics Workbench v 6.5.1 (CLC Bio, Aarhus, Denmark), quality trimming (limit = 0.05, maximum number of ambiguous nucleotides = 2) read mapping (minimum length fraction = 0.8, minimum similarity fraction = 0.8, random matching allowed, otherwise default settings) of each sample individually against the potato reference genome pseudomolecule model (DM version 4.03 available at http://solanaceae.plantbiology.msu.edu/pgsc_download.shtml) (Potato Genome Sequencing Consortium et al. 2011; Sharma et al. 2013). Resulting mappings were exported in the BAM format and converted into tab-separated text files reporting the base/coverage distribution at each position using an in-house perl script (BAM2coverage.pl). Hereafter, quality-based variant detection was performed on both parents (HAG and HAF), and low and high WUE bulks separately using the following criteria: broken pairs were ignored (to avoid spurious variants due to erroneous mapping of the reads); the presence of both forward and reverse reads was required; minimum total coverage of 10 reads; minimum variant frequency = 35 %; and the maximum expected allele number was set to 2 (both parents are diploidal). Detected variants were exported into.csv format, combined and filtered to only include SNPs observed in either of the parents and with a minimum coverage of 10 in both parents and bulks using in-house perl scripts (CreateMarkerFile.pl and AddBulkAndPvalue.pl).

### QTL and statistical analysis

At each SNP marker position, the weighted average of each base distribution from parental mappings on the chromosomes was reported, hereby creating a list of markers, where the parental generation displays heterozygosity. Following, the parental base distribution was compared with the base distribution of each bulk by combining Fisher’s exact test (Eq. 1) and Fisher’s method (Eq. 2) reporting a *p* value (by converting *χ*^2^ statistics using 8 degrees of freedom).

Equation 1: Fisher’s exact test equation, where *p* is a *p* value for each base (X = A, T, G or C) coming from comparison of parental base distribution (exp) and bulk base (either low or high bulk) distribution (obs). The total coverage at base position is designated with *n*.

1 $$ p_{X}  = \frac{{\left( {\begin{array}{*{20}c}    {\exp (n = X) + {\text{obs}}(n = X)}  \\    {\exp (n = X)}  \\   \end{array} } \right)\left( {\begin{array}{*{20}c}    {\exp (n \ne X) + {\text{obs}}(n \ne X)}  \\    {\exp (n \ne X)}  \\   \end{array} } \right)}}{{\left( {\begin{array}{*{20}c}    {{\text{ }}n}  \\    {\exp (n = X) + \exp (n \ne X)}  \\   \end{array} } \right)}}. 
$$

Equation 2: Fisher’s method equation based on Chi-square distribution with 2 k degrees of freedom (8 = 2 × 4 different bases—A,T,G,C), where *k* is a number of tests being combined.

2 $$ \chi_{2k}^{2} = - 2\left( {\ln (p_{A} ) + \ln (p_{T} ) + \ln (p_{G} )\ln (p_{C} )} \right). $$

Using a Bonferroni corrected significance threshold of 0.001, the relative skewness [*RS*] between significant and non-significant markers was calculated in window sizes of 1 Mb in step of 0.1 Mb along each chromosome (by use of perl script CalculateSkewness.pl) according to Eq. 3.

Equation 3: Relative skewness calculation, where RS_i_ is each *i*th bin relative contribution to overall positive skewness. Ratio of significant markers to all markers in a bin is designated as *x*_i_, whereas *x*_total_ is an overall ratio of all significant markers to all markers at each chromosome.

3 $$ RS_{\text{i}} = (x_{\text{i}} - x_{\text{total}} )^{3} . $$

In regions with total number of markers less than 20, the relative skewness was set to 0. The relative skewness was plotted along the chromosome, hereby identifying regions with high density of significant SNPs, i.e., non-randomly selected alleles in the bulks. All perl scripts are available in supporting material.

### Glutamine synthetase activity assay

Leaf samples were taken at two time points in 2010 (21 and 25 clones of low and high WUE bulks, respectively) and at two points in 2011 (11 clones in low WUE bulk and 13 clones of high WUE bulk). At each of these time points, environmental conditions were different, namely: *T* = 28 °C, *Rh* = 85 %, light = 1200PAR, [CO_2_] = 380 ppm (16/08/2010); *T* = 20 °C, *Rh* = 85 %, light = 1200PAR, [CO_2_] = 1000 ppm (30/08/2010); *T* = 32 °C, *Rh* = 50 %, light = 1200PAR, [CO_2_] = 380 ppm (13/05/2011); and *T* = 28 °C, *Rh* = 80 %, light = 700PAR, [CO_2_] = 1000 ppm (27/05/2011). 100–150 mg of leaf tissue was homogenized in a Precellys CK14 tissue homogenization tube (including beads) and subjected to three cycles of 15 s (with 10 s pause in between) homogenization at 5000 rpm using a Precellys mechanical homogenizer (Bertin Technologies, France). Disrupted tissue was transferred to a 2-ml microcentrifuge tube and mixed with 400 ml of extraction buffer (20 mM Tris–HCl pH 8.0, 4 mM DTT, 5 mM MgCl_2_) followed by centrifugation at 14,000 rpm for 5 min. The supernatant was transferred to a new tube and subjected to a second centrifugation. The resulting supernatant was subsequently used for a glutamine synthetase (GS) activity assay performed as described by Kingdon et al. (1967) with several modifications (Kingdon et al. 1967). 4 ml of reaction mixtures containing 0.1 M l-glutamine, 2.5 mM sodium arsenate, 0.225 mM MnCl_2_, 6.25 mM hydroxylamine, 0.125 mM adenosine disphosphate (ADP), and either 10 % v/v enzyme extract for samples or 1 mM Tris–HCl buffer for blanks was incubated for 30 min at 37 °C. Reactions were stopped by adding 1 ml of ferric chloride reagent (prepared by dissolving 10 g of trichloro-acetic acid and 8 g of ferric chloride in 250 ml of 0.5 N hydrochloric acid). Developed brown color was measured at 540 nm by use of Tecan Infinite M1000 (Tecan, Mannedorf, Switzerland) spectrophotometer.

## Results

### WUE phenotyping

**Fig. 1:**
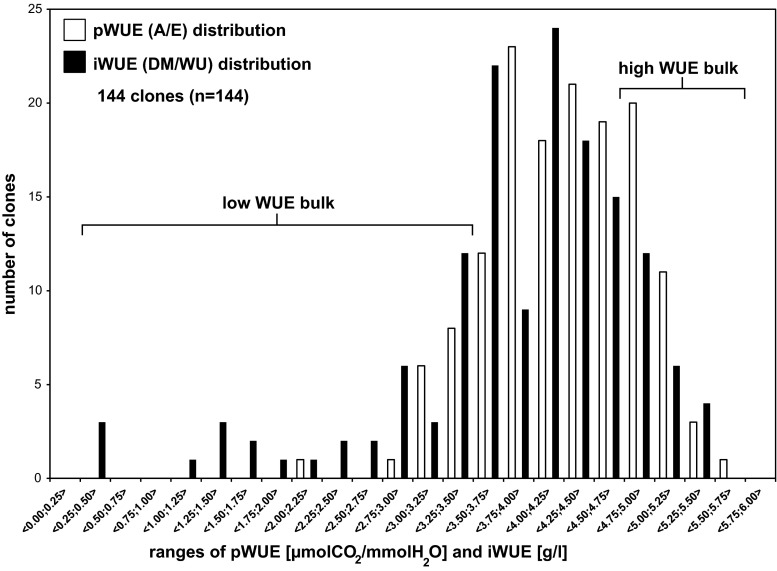
Histogram of photosynthetic WUE (*pWUE*) and irrigation (*iWUE*) distribution for the mapping population in 2010. A total of *n* = 144 clones were phenotyped. 54 independent measurements of *pWUE* and 2 measurements of *iWUE* were performed. *Y*-axis corresponds to the number of clones that are in a specific range on *x*-axis of *pWUE* (µmolCO_2_/mmolH_2_O) and *iWUE* (g/l). Clones of low and high WUE bulk are indicated by brackets

The mapping population of 144 clones together with two parental lines was phenotyped for photosynthetic WUE (*pWUE*) and irrigation WUE (*iWUE*) (see “Methods” sections for details). As seen in Fig. 1, pWUE appears normally distributed, whereas iWUE appears normally distributed but negatively skewed with clones of low iWUE. Based on this, two bulks of clones were made, a low bulk (21 clones) and a high bulk (24 clones), as indicated in Fig. 1. The average values of rate of photosynthesis (A), rate of transpiration (E), and resulting pWUE (A/E) as well as dry matter (DM), water use (WU), and resulting iWUE (DM/WU) are shown in Table 1. When comparing high and low bulks using Student’s *t* test, the high bulk showed significantly higher *A* (34 %, *p* value = 7.0 × 10^−36^) and DM (61 %, *p* value = 2.8 × 10^−4^). No significant differences could be observed in *E*; however, WU was significantly elevated in the high bulk by 20 % (*p* value = 1.1 × 10^−2^). Interestingly, as a product of all single parameters, pWUE and iWUE were also significantly increased (35 %, *p* value = 3.9 × 10^−35^ and 4.4 × 10^−5^, respectively).

**Table 1:** Phenotypic parameters of WUE bulks

Parameter	Low	High
*A* (µmolCO_2_/m^2^s)	7.39 ± 0.13*	9.87 ± 0.14*
*E* (mmolH_2_O/m^2^s)	2.07 ± 0.03	2.03 ± 0.03
pWUE (A/E) (µmolCO_2_/mmolH_2_O)	3.58 ± 0.09*	4.85 ± 0.10*
DM (g)	24.3 ± 1.3*	39.1 ± 1.0*
WU (l)	7.1 ± 0.2*	8.5 ± 0.1*
iWUE (DM/WU) (g/l)	3.4 ± 0.2*	4.6 ± 0.1*

(A) Photosynthesis (µmolCO_2_/m^2^s), (E) transpiration (mmolH_2_O/m^2^s), (pWUE) photosynthetic WUE (µmolCO_2_/mmolH_2_O), (DM) dry matter (g), (WU) water use (l), and (iWUE, DM/WU) irrigation WUE (g/l). Asterisk indicates a significant difference at *p* < 0.05

### Sequencing and reference mapping

DNA was extracted for all 47 samples (21 for low WUE bulk, 24 for high WUE bulk and 2 parents), and the resulting DNA was pooled stoichiometrically prior to sequencing. Sequencing resulted in approximately 51 and 46 billion bases (after quality trimming) for HAF and HAG, respectively; and 49 and 70 billion bases for the low and high WUE bulks, respectively. Reads were mapped to the potato genome pseudomolecule model (v4.03). Mapping metrics is shown in Table 2. The number of mapped bases was similar for the parental lines and bulks and exceeded 87 %. For all mappings, fraction of the reference genome covered was higher than 0.84 (84 %, Table 2). The average coverage over consensus sequences ranges from ×56 for HAG to ×80 for the high bulk (Table 2).

**Table 2:** Reference mapping statistics of both parents (HAF, HAG) together with low and high WUE bulks

Mapping	Total bases	Mapped bases	% of mapped bases	Coverage (Fraction)
90-HAF-01	51,248,139,187	46,038,419,272	89.83	59.97 (0.85)
90-HAG-15	49,778,099,796	43,481,825,907	87.35	56.44 (0.84)
Low	49,056,521,511	44,944,643,704	91.62	58.68 (0.85)
High	70,636,394,226	62,032,158,617	87.82	79.81 (0.85)

### Quality-based variant detection

As the next step of the bioinformatic analysis, quality-based variant detection was performed for both parental lines. Variants included not only single nucleotide variations (SNVs) but also multi nucleotide variations (MNVs) as well as insertions/deletions (InDels), which can potentially be used for downstream analysis. For HAF 9,020,103 and for HAG 12,038,807, variations were detected. Subsequently, a marker list was created with SNVs (SNPs) only originating from parental variant detection and comprising coverage from weighted average of both parents. As a result, 9,144,431 SNPs were included in the marker list, giving an average of 1SNP per 83 bp, entirely sufficient for QTL analysis. The resulting combined marker list with parental coverage was then statistically compared with coverage distribution of low and high WUE bulks separately.

### QTL analysis

**Fig. 2:**
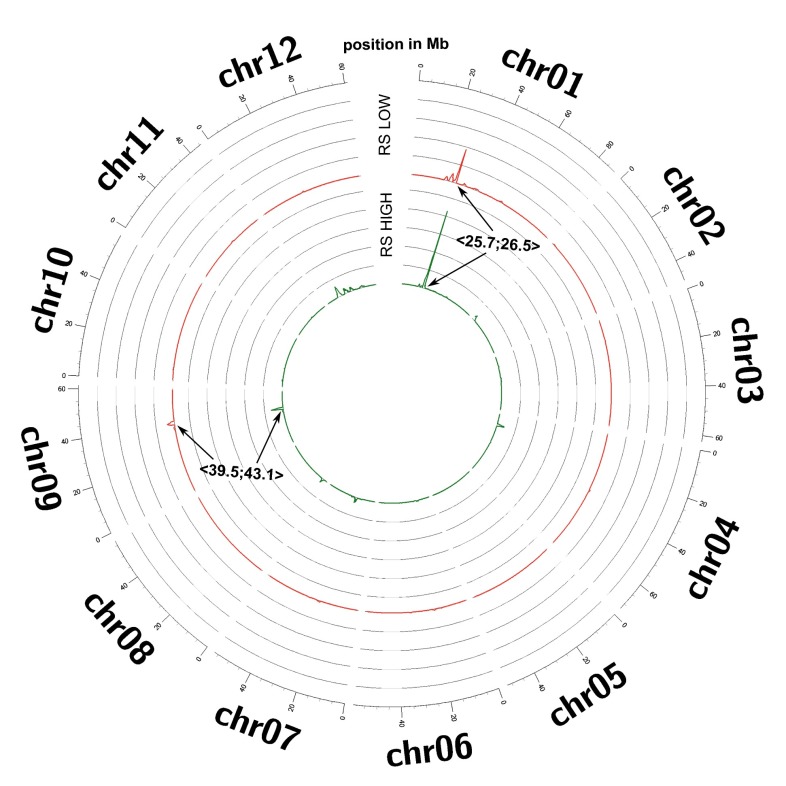
Relative skewness measure (all scaled to the same value of relative contribution to skewness) of low and high bulk mapping marker distribution on 12 reference potato genome chromosomes. Sliding window analysis was performed with bin size of 2 Mb and step size of 0.2 Mb. Two overlapping peaks in low and high bulks were detected, at chr01 〈24.7;27.5〉 and at chr09 〈39.5;43.1〉

A comparison between parental base distribution and low and high WUE bulks distribution was performed at each SNP marker position. The test used was a combined Fisher’s exact test returning *p* value for each SNP position (see methods for details). SNPs were considered significant when the Bonferroni corrected *p* value was lower than 0.01 %. A relative skewness measure of resulting peaks which identified QTLs of interest was visualized by a sliding window analysis (see Fig. 2). Assuming that it is the same QTL(s) that distinguish and are responsible for the observed phenotypic difference of the low and high WUE bulks in the mapping population, only common peak regions were subjected to further detailed analysis. Two QTLs were found: a larger at chromosome 1: 〈24.7;27.5〉 (Mb) (73 genes under the peak) and a smaller at chromosome 9: 〈39.5;43.1〉 (Mb) (105 genes under the peak). Genes of immediate interest, e.g., genes that could intuitively be associated with photosynthesis and WUE, could not be found at chromosome 9, but were identified at chromosome 1 and are listed in Table 3. Interestingly, they comprise three genes (PGSC0003DMG403009595, PGSC0003DMG402009595, and PGSC0003DMG401009595) encoding GS, an enzyme essential for nitrogen fixation and mobilization that can directly be associated with photosynthesis through the photorespiratory carbon and nitrogen cycle. One gene, PGSC0003DMG402009595, seems to be truncated and thus is unlikely to be functional. A full list of genes enclosed within peaks found in both the low and high pool can be found in Supporting Table 1. In the entire potato genome, there appears to be 9 genes encoding full length GS isoforms (Supporting Table 2). Following peptide sequence alignment, several deeply separated clades (Supporting Fig. 1) can be identified. Similar to *Arabidopsis thaliana*, a single gene is encoding the plastid glutamine synthetase (GS2) and a clade of cytosolic glutamine synthetases (GS1) comprising 5 genes. The two full length genes under the QTL are members of this clade and contain no detectable signal sequences [SignalP 4.1 (Petersen et al. 2011), TargetP 1.1 (Emanuelsson et al. 2000) and WoLF PSORT (Horton et al. 2007)] as expected for a cytoplasmic protein.

**Table 3:** List of candidate glutamine synthetase genes

Region (bp)	ID	Coverage				Expression (FPKM)—leaf	
		HAF	HAG	Low	High	DM1-3 516 R44	RH89-039-16
27,407,755–27,416,055	PGSC0003DMG403009595	24.4	21.3	24.5	29.23	5.19	2.29
27,437,064–27,445,056	PGSC0003DMG402009595	31.5	37.5	43.8	65.29	4.73	0
27,452,241–27,457,303	PGSC0003DMG401009595	26.9	48	45.6	55.79	2.12	1.48

Gene expression data as fragment per kilobase of exon per million fragments mapped (FPKM) of *Solanum tuberosum* ssp*. tuberosum* (RH89-039-16) and the *Solanum tuberosum* ssp. *phureja* (DM1-3 516 R44) come from potato reference genome analysis (Chen and Setter 2012)

The expression of the genes of the cytosolic glutamine synthetase clade was determined for a number of tissues for the *Solanum tuberosum* ssp. *tuberosum* (RH89-039-16 (RH)) and the *Solanum tuberosum* ssp. *phureja* [DM1-3 516 R44 (DM)] as part of the potato genome sequencing project (Potato Genome Sequencing Consortium et al. 2011). In DM, all three genes found under the peak at chr01 are expressed, PGSC0003DMG403009595 and PGSC003DMG402009595 roughly twice as high as PGSC0003DMG401009595. In RH, transcripts from PGSC003DMG402009595 (the truncated version) were not detected, but again PGSC0003DMG403009595 is slightly higher expressed than PGSC0003DMG401009595 (1.5 fold), see Supporting Table 2. Analyzing expression of our two candidate genes (PGSC0003DMG401009595 and PGSC0003DMG403009595), as seen in Supporting Table 2, they constitute significant part of overall cytosolic GS1 clade expression (from DM1-3 516 R44 and RH89-039-16 expression). By excluding gene PGSC0003DMG400004355, which is clearly plastidic version (see Supporting Fig. 1), our two candidates constitute 14.66 % of overall expression, but 53.14 % when right part of GS1 clade is taken alone.

### Glutamine synthetase

**Fig. 3:**
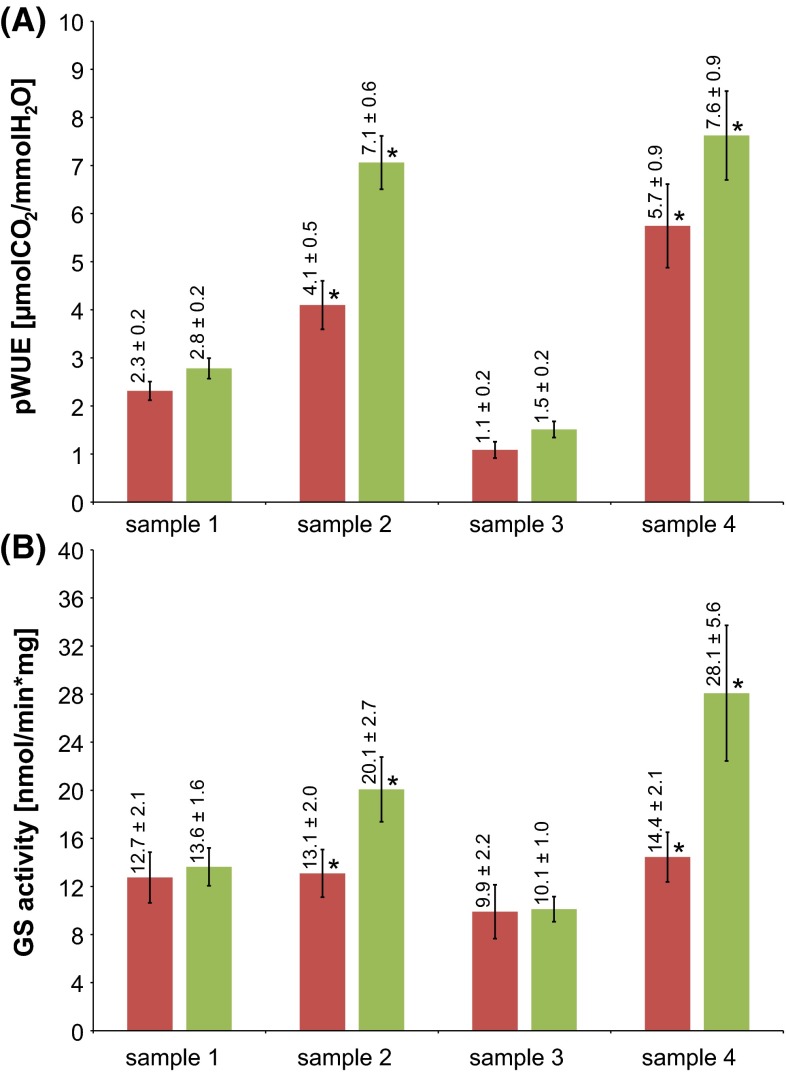
GS activity [nmol/min × mg] and *pWUE* (µmolCO_2_/mmolH_2_O) at four different samples: sample 1—16/08/2010 (*T* = 28 °C, *H* = 85 %, light = 1200PAR, [CO_2_] = 380 ppm), sample 2—30/08/2010 (*T* = 20 °C, *H* = 85 %, light = 1200PAR, [CO_2_] = 1000 ppm), sample 3—13/05/2011 (*T* = 32 °C, *H* = 50 %, light = 1200PAR, [CO_2_] = 380 ppm), and sample 4—27/05/2011 (*T* = 28 °C, *H* = 80 %, light = 700PAR, [CO_2_] = 1000 ppm). Figure indicates GS activity and pWUE values together with standard errors above corresponding bars. *Red color* indicates low WUE bulk, whereas* green color* high WUE bulk. Significant difference at the same time point between low and high bulks is indicated with an* asterisk* (*)

To validate that the differential use of alleles of GS translates into different GS enzyme activity, extracts from leaf samples at four time points of contrasting environmental conditions were analyzed using a GS enzyme activity assay (Fig. 3). Representative samples were taken from four different environmental conditions in two different years on independent plants. As can be seen from Fig. 3, conditions where pWUE is significantly different (at high CO_2_ concentrations) between low and high WUE bulks, the enzyme activity of total GS activity is also significantly different in agreement with the importance of the candidate genes identified.

**Fig. 4:**
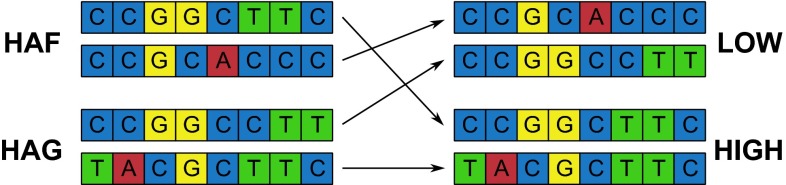
Allelic segregation between low and high WUE bulks based on the base distribution at SNVs of glutamine synthetase (PGSC0003DMG403009595). The four alleles segregate into low bulk: CCGCACCC (coming from HAF) and CCGGCCTT (from HAG) and high bulk: CCGGCTTC (from HAF) and TACGCTTC (from HAG)

The genes encoding GS were further analyzed according to their amino acid (AA) changing mutations in reference to the potato genome model. All AA changing SNPs within GS genes were identified and are presented in Table 4. Only one of the glutamine synthetase genes (PGSC0003DMG403009595) had AA changing SNPs. Gene diagram is presented in Supporting Fig. 2, where AA changing SNPs span over 4 exons. From the parental coverage distribution presented in Table 4, it can be deduced that the gene had two alleles per parent (HAF: CCGGCTTC and CCGCACCC and HAG: CCGGCCTT and TACGCTTC using AA changing SNVs as notation). Also the allelic segregation between low and high WUE bulks based on the base distribution can be suggested. The four alleles segregate into low bulk: CCGCACCC (coming from HAF) and CCGGCCTT (from HAG) and high bulk: CCGGCTTC (from HAF) and TACGCTTC (from HAG), as indicated in Fig. 4. The proposed allelic segregation is further supported by all reads mapped to the reference genome (data not shown). According to PFAM domain search employed by CLC workbench, all AA changing SNPs detected belonged to glutamine synthetase C-terminal catalytic domain PF00120.19 which position was predicted to span from 30 to 235 amino acid.

**Table 4:** List of SNPs resulting in amino acid change at genes of glutamine synthetase (PGSC0003DMG403009595)

Position	HAF				HAG				Low				High				Mutation	Amino acid change
	A	T	G	C	A	T	G	C	A	T	G	C	A	T	G	C		
27,408,203	0	0	0	50	0	36	0	47	0	5	0	72	0	28	0	44	C>T	Ala222Thr
27,408,239	0	0	0	47	47	1	0	45	3	0	0	75	40	0	0	49	C>A	Val210Leu
27,408,271	0	0	54	0	0	0	50	53	0	0	75	4	0	1	50	38	G>C	Ala199Gly
27,408,292	0	0	24	28	0	0	90	16	0	0	44	20	0	0	61	20	C>G	Cys192Ser
27,408,329	16	0	0	17	11	0	1	82	24	0	0	15	13	0	0	62	C>A	Ala180Ser
27,408,745	0	31	0	23	0	29	0	30	0	9	0	43	0	26	0	58	T>C	Asn130Asp
27,410,234	0	29	0	20	0	53	0	23	0	52	0	57	0	49	0	35	C>T	Val111Ile
27,415,438	0	0	0	38	0	18	0	29	0	43	0	48	0	36	0	72	C>T	Ala69Thr

## Discussion

A reciprocal cross of diploidal potato lines of similar phenotype produced approximately normally distributed mapping population in respect to both *pWUE* and *iWUE*, with the parents of moderate phenotype. Phenotyping of pWUE was performed in 54 different environmental conditions to ensure detection of core/universal QTL by minimizing negative effect of genotype × environment interaction. This indicated an expected polygenetic origin of this trait as well as a genetic potential for improving this trait. Bulk segregation was based on measurements of *pWUE* and *iWUE* and bulks of low (21 clones) and high (25 clones) WUE were identified. These may appear relatively large in respect to total size of the mapping population (144), but large bulks maximizes the power of the QTL detection despite of weaker selection and allele frequency differences, in sequence based approaches as previously described by Magwene et al. (2011). Furthermore, they found that increasing sequence coverage is only increasing the ability to detect bulk dependent differences until the sequence coverage exceeds the number of individuals in the bulk (Magwene et al. 2011). We have nonetheless ensured that coverage should at least be equal to the number of individuals in the bulk multiplied by ploidy of the mapping population.

The low WUE bulk was sequenced to ×59 coverage and consisted of 21 clones, whereas high bulk was sequenced to ×80 coverage and consisted of 24 clones. Since there is an abundance of polymorphic sites, there was no need to include other types of variation than SNVs. Worthy of note, the method employed does not require any prior knowledge about molecular markers because their position and base coverage distribution result solely from genome re-sequencing of the parents. Such QTL analysis can therefore in principle be used for any phenotyped and sampled mapping population for which parental sequences can be obtained and a reference genome is available.

9,144,431 SNPs were used for the QTL analysis. This corresponds to a variation of 1 SNP per 83 bp, which is a relatively large genetic variation. Few of the determined SNPs are likely to occur due to sequencing errors, a result of erroneous mapping or from errors in the reference genome sequence. Nonetheless, the resulting de novo marker file served as the sole source of markers by following the assumption that the maximal number of markers detected in the mapping population is a reflection of two diploidal parents used to produce a cross. Therefore, the sequence data of the bulks were not used in marker determination. Using Fisher’s exact test, comparing the weighted average of the parental distribution at each SNP position with the observed base distribution of each bulk separately, it was possible to identify non-random variations associated with the selection of individuals in the bulk. From this it was possible to delimit relatively narrow regions of the genome containing candidate genes of interest. We have also employed Fisher’s *χ*^2^ test with very similar results (data not shown). In fact, the *χ*^2^ test is easier to compute for higher sample sizes (in our case, read coverage). Under the assumption that it is the same QTL(s) which is responsible for the observed phenotypic difference separating both the low and high group from the average of the population, they can serve as each other’s controls. In both analyses, QTLs were found on chromosome 1 and 9.

Under all peaks identified, 178 genes were found, of which no less than 129 genes were annotated with unknown function, and a number of genes with only a very general function assigned were present, see Supporting Table 1. We cannot rule out that these genes contribute to the QTLs. However, it was immediately interesting that the QTL on chromosome 1 contains a cluster of genes encoding glutamine synthetase (GS). Indeed, determining the total GS enzyme activity of selected samples shows that under conditions where pWUE is significantly higher in the high group compared to the low group, the GS enzyme activity is also significantly higher. Taken together that allelic differences separate the high and low group at the candidate loci only, this strongly supports the identified GS genes involvement in *pWUE*.

Glutamine synthetase (GS) is a key enzyme in nitrogen metabolism and is responsible for ATP-dependent ammonium (NH_4_^+^) fixation to the δ-carboxyl group of glutamate to form the amino acid glutamine. GS plays a crucial role not only in plant growth by assimilating ammonium from the soil, but also in the re-assimilation of photorespiratory ammonium released during photorespiration, which can exceed primary nitrogen assimilation by as much as 10-fold (Keys et al. 1978). GS has been shown to play role in WUE by being responsive to drought and salt stress (Bernard and Habash 2009). In all higher plants, two distinct isoenzymes of glutamine synthetase are present: cytosolic (GS1) and chloroplast (GS2) (Cock et al. 1991; Oliveira and Coruzzi 1999; Peterman and Goodman 1991; Sakakibara et al. 1992; Sakamoto et al. 1990; Tingey and Coruzzi 1987). GS2 is the major isoenzyme in leaves, and mutants deficient in GS2 activity were shown to decline photorespiration and increase ammonium concentrations significantly, therefore, causing significant decrease in photosynthesis in barley (Blackwell et al. 1987; Wallsgrove et al. 1987). Barley plants with decreased expression of GS2 have also shown not to be able to grow under photorespiratory conditions (Wallsgrove et al. 1987). Overexpression of GS2 on the other hand proved to increase photorespiratory capacity in wheat (Palatnik et al. 1999) and rice grown under osmotic stress (Hoshida et al. 2000). While GS2 was shown in most plant species to be encoded by a single gene (Becker et al. 1992; Lightfoot et al. 1988), cytosolic GS1 is encoded by multiple homologous genes (Cock et al. 1991; Oliveira and Coruzzi 1999; Peterman and Goodman 1991; Sakakibara et al. 1992; Sakamoto et al. 1990; Tingey and Coruzzi 1987). In contrast to the above studies, our candidate genes are cytosolic variant (GS1) of GS. Even though this may immediately seem puzzling, overexpression of GS1 has previously been shown to increase photosynthesis and growth, and decrease free ammonium levels in leaves (Fuentes et al. 2001; Oliveira et al. 2002). This points toward that effective re-assimilation of the volatile NH_3_ is dependent on both chloroplastic and cytosolic GS, possibly because NH_3_ can be transported into the cytosol either passive diffusion across or facilitated via aquaporins present in the chloroplast membrane (Maurel et al. 2008). Indeed, plastid GS2 is likely also important for WUE, but might very well not be detected in our analysis simply because we have no functional variance of this loci present in the population analyzed. According to Fig. 3, significant differences in *pWUE* and GS activity were observed in samples, where the carbon dioxide concentration [CO_2_] was elevated to 1000 ppm, approximately two and a half times higher than the current atmospheric concentration. Such GS stimulation has been previously reported in, e.g., sunflower (Larios et al. 2004) or in carob (Cruz et al. 2003). GS2 and GS1 were also both shown to be induced by light or by carbon metabolites (mainly sucrose) (Edwards and Coruzzi 1989; Oliveira and Coruzzi 1999). Since one of the most apparent effects of plants response to elevated [CO_2_] is the increase in non-structural carbohydrates (Ainsworth and McGrath 2010), a higher overall GS stimulation under high [CO_2_] (sample 2 and sample 4), compared to ambient [CO_2_] (sample 1 and sample 3), is expected. Interestingly, the increase of GS activity is much higher in the high WUE bulk than in the low bulk under elevated [CO_2_], indicating a higher responsiveness of GS in the high bulk caused by the differential allelic distribution between the two groups. Up to this point, only one atomic structure of plant glutamine synthetase has been determined (Unno et al. 2006). Interestingly, they have identified several key residues in maize cytosolic GS responsible for stability and activity of GS that can be correlated to our AA changing SNPs in Table 4. These residues are isoleucine and serine that are located in similar region of catalytic C-domain as two of AA changing SNPs in Table 4. Isoleucine (Ile) residue was found to be essential for GS heat stability, whereas serine is part of catalytic center that binds ADP (Unno et al. 2006). Two SNPs from Table 4, Val111Ile and Ala180Ser, point into conclusion that high bulk contains predominantly Ile and Ser residues suggesting functional alleles. Reliable conclusions about functionality of alleles identified in this study would, however, require further analysis, most probably determination of their atomic structure. Considering that the observed difference in WUE and the associated difference in GS only manifest itself under such high CO_2_ concentrations might suggest that this is not relevant at normal conditions. However, these experiments were carried out under well-watered and well-fertilized conditions where N cannot be expected to be limited unless under the extreme rapid growth caused by high CO_2_ conditions. Assimilation of nitrogen requires concurrent carbon uptake, and therefore, there is tight correlation between N and C metabolism (Thomsen et al. 2014). As a consequence, insufficient C supply (as in our case under normal CO_2_ conditions) will reduce the positive effects of functional GS1 if N is otherwise supplied (as in this case under well-fertilized and watered conditions under high [CO_2_]). NH_4_+ is constantly generated in high amounts in leaf tissue due to photorespiration, nitrate reduction, or protein turnover. Photorespiratory N cycling, however, may be 10 times higher than primary N assimilation. The role of cytosolic GS1 in refixation of NH_3_ is therefore of utmost importance to avoid NH_4_+ accumulation and loss of NH3 from the leaf tissue due to emission (plant-atmosphere ammonia exchange) (Schjoerring et al. 2000). Under field conditions, the availability of N is much lower, and thus, the importance of limiting the loss of N due to photorespiration can be expected to be important even at ambient CO_2_ conditions. However, this remains to be shown.

For the first time, successful phenotyping of a trait as complex as WUE in a potato mapping population of different genotypes was performed, and somewhat surprisingly, cytosolic GS genes important for limiting N loss due to photorespiration were identified as important QTLs for this trait. Arguably, under the applied well-watered and well-fertilized conditions, these QTLs may be considered a QTL for rapid growth rate rather than for WUE.

### Author contribution statement

K.P.K.—main research and project development, performance of all experiments and biostatistical analysis; K.K. & M.N.A.—construction of the phenotyping experiment and interpretation of the WUE results; M.S. & M.S.A.—pipeline development for bioinformatic analysis, construction of the statistical analysis; H.G.K.—creation of mapping population at breeding station; K.L.N.—main project supervisor, author of the QTL analysis method.

## Electronic supplementary material

Supplementary material 1 (PDF 95 kb)

Supplementary material 2 (PDF 162 kb)

Supplementary material 3 (DOCX 25 kb)

Supplementary material 4 (DOCX 15 kb)
