# Supplementary figures and images for: Cytosolic glutamine synthetase is important for photosynthetic efficiency and water use efficiency in potato as revealed by high-throughput sequencing QTL analysis
Source: Theor Appl Genet. 2015 Jul 12;128(11):2143–53. doi: 10.1007/s00122-015-2573-2 (PMC4624824; doi:10.1007/s00122-015-2573-2)

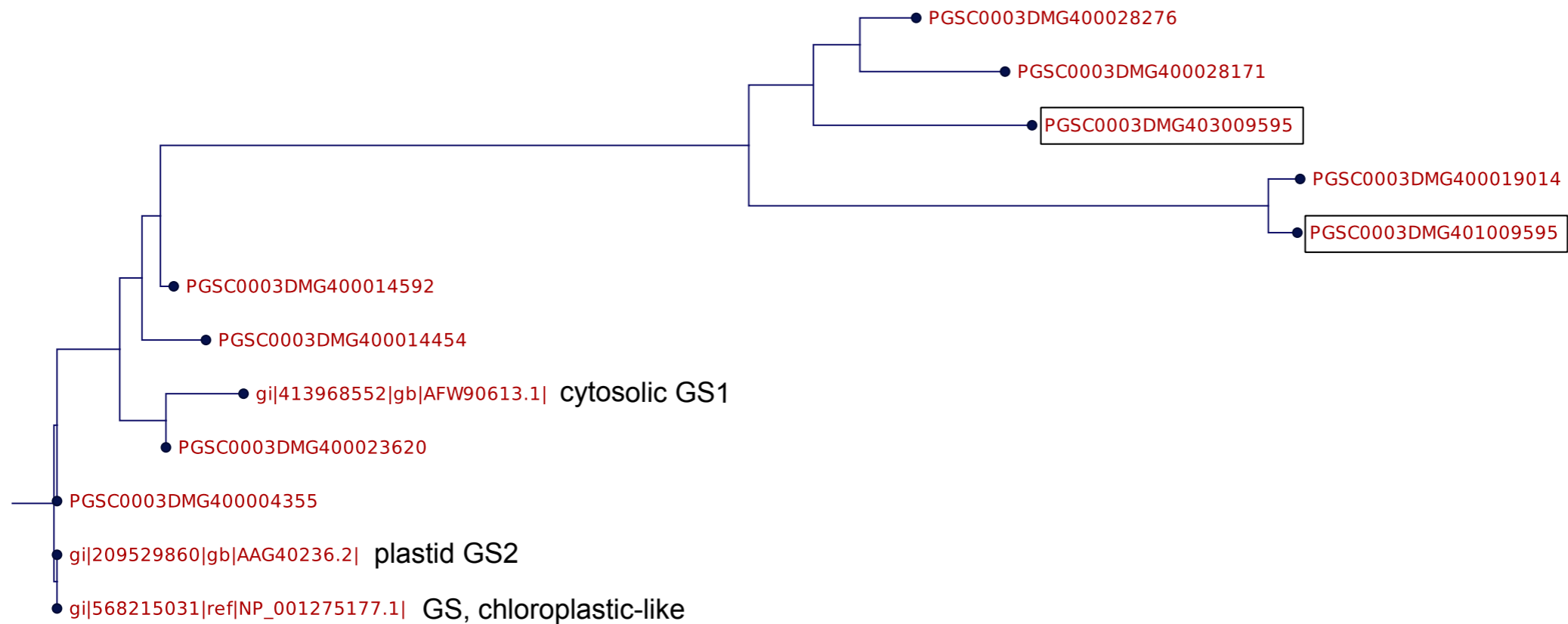

1.500

Supplement: Supplementary file 1 — Supplementary material 1 (PDF 95 kb) [file 122_2015_2573_MOESM1_ESM.pdf]
