# Supplementary material for: Cytosolic glutamine synthetase is important for photosynthetic efficiency and water use efficiency in potato as revealed by high-throughput sequencing QTL analysis
Source: Theor Appl Genet. 2015 Jul 12;128(11):2143–53. doi: 10.1007/s00122-015-2573-2 (PMC4624824; doi:10.1007/s00122-015-2573-2)

1) Pos. 27408203 C>T ALA222Thr 2) Pos. 27408239 C>A Val210Leu 3) Pos. 27408271 G>C Ala199Gly 4) Pos. 27408292 C>G Cys192Ser 5) Pos. 27408329 C>A Ala180Ser 6) Pos. 27408745 T>T Asn130Asp 7) Pos. 27410234 C>T Val111Ile 8) Pos. 27415438 C>T Ala69Thr

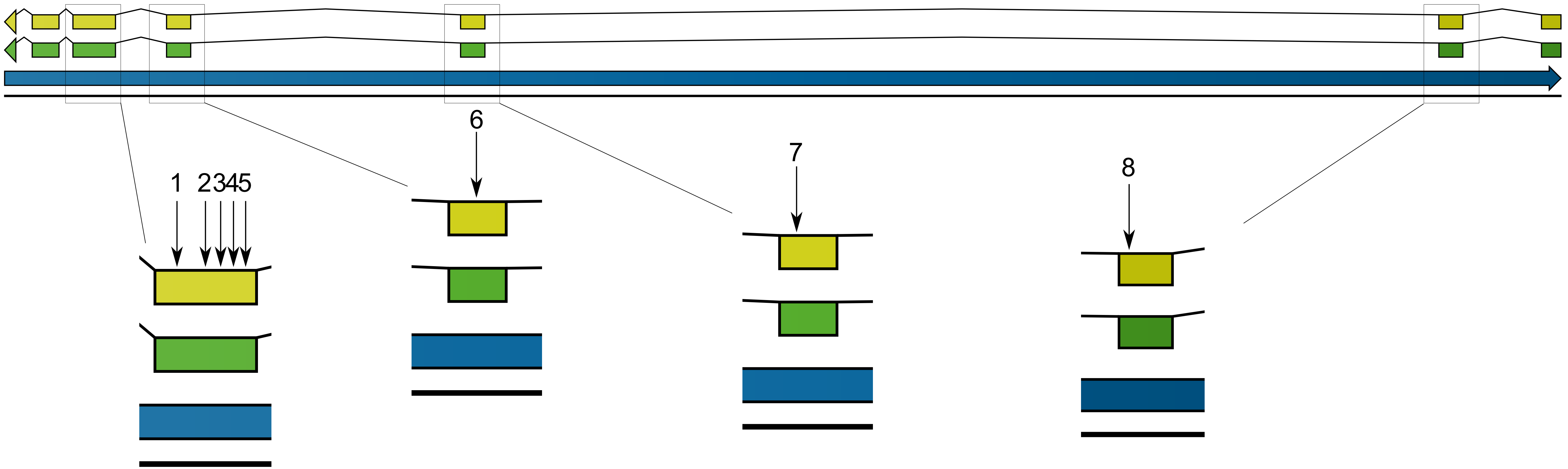

Supplement: Supplementary file 2 — Supplementary material 2 (PDF 162 kb) [file 122_2015_2573_MOESM2_ESM.pdf]
