# Supplementary material for: Cytosolic glutamine synthetase is important for photosynthetic efficiency and water use efficiency in potato as revealed by high-throughput sequencing QTL analysis
Source: Theor Appl Genet. 2015 Jul 12;128(11):2143–53. doi: 10.1007/s00122-015-2573-2 (PMC4624824; doi:10.1007/s00122-015-2573-2)
Supplement: Supplementary file 3 — Supplementary material 3 (DOCX 25 kb) [file 122_2015_2573_MOESM3_ESM.docx]

| Name | Chromosome | Qualifiers |
| --- | --- | --- |
| PGSC0003DMG400035395 | 1 | /source=BGI; /ID=PGSC0003DMG400035395; /name=Gene of unknown function |
| PGSC0003DMG400037526 | 1 | /source=BGI; /ID=PGSC0003DMG400037526; /name=Gene of unknown function |
| PGSC0003DMG400040858 | 1 | /source=BGI; /ID=PGSC0003DMG400040858; /name=Gene of unknown function |
| PGSC0003DMG400036483 | 1 | /source=BGI; /ID=PGSC0003DMG400036483; /name=Gene of unknown function |
| PGSC0003DMG400043241 | 1 | /source=BGI; /ID=PGSC0003DMG400043241; /name=Gag-pol polyprotein |
| PGSC0003DMG400044613 | 1 | /source=BGI; /ID=PGSC0003DMG400044613; /name=Gene of unknown function |
| PGSC0003DMG400037680 | 1 | /source=BGI; /ID=PGSC0003DMG400037680; /name=Gene of unknown function |
| PGSC0003DMG400035392 | 1 | /source=BGI; /ID=PGSC0003DMG400035392; /name=Gene of unknown function |
| PGSC0003DMG400039964 | 1 | /source=BGI; /ID=PGSC0003DMG400039964; /name=Gene of unknown function |
| PGSC0003DMG400043668 | 1 | /source=BGI; /ID=PGSC0003DMG400043668; /name=Gene of unknown function |
| PGSC0003DMG400040680 | 1 | /source=BGI; /ID=PGSC0003DMG400040680; /name=Gene of unknown function |
| PGSC0003DMG400036254 | 1 | /source=BGI; /ID=PGSC0003DMG400036254; /name=Gene of unknown function |
| PGSC0003DMG400044915 | 1 | /source=BGI; /ID=PGSC0003DMG400044915; /name=Gene of unknown function |
| PGSC0003DMG400028869 | 1 | /source=BGI; /ID=PGSC0003DMG400028869; /name=Prolyl endopeptidase |
| PGSC0003DMG400037871 | 1 | /source=BGI; /ID=PGSC0003DMG400037871; /name=Conserved gene of unknown function |
| PGSC0003DMG400045561 | 1 | /source=BGI; /ID=PGSC0003DMG400045561; /name=Gene of unknown function |
| PGSC0003DMG400041954 | 1 | /source=BGI; /ID=PGSC0003DMG400041954; /name=(Fragment) |
| PGSC0003DMG400040478 | 1 | /source=BGI; /ID=PGSC0003DMG400040478; /name=Gene of unknown function |
| PGSC0003DMG400036850 | 1 | /source=BGI; /ID=PGSC0003DMG400036850; /name=Gag-pol polyprotein |
| PGSC0003DMG400046530 | 1 | /source=BGI; /ID=PGSC0003DMG400046530; /name=Integrase core domain containing protein |
| PGSC0003DMG400037267 | 1 | /source=BGI; /ID=PGSC0003DMG400037267; /name=Gene of unknown function |
| PGSC0003DMG400046132 | 1 | /source=BGI; /ID=PGSC0003DMG400046132; /name=Gene of unknown function |
| PGSC0003DMG400042947 | 1 | /source=BGI; /ID=PGSC0003DMG400042947; /name=Axi 1 protein |
| PGSC0003DMG400038198 | 1 | /source=BGI; /ID=PGSC0003DMG400038198; /name=Gene of unknown function |
| PGSC0003DMG400046466 | 1 | /source=BGI; /ID=PGSC0003DMG400046466; /name=Gene of unknown function |
| PGSC0003DMG400040411 | 1 | /source=BGI; /ID=PGSC0003DMG400040411; /name=Gene of unknown function |
| PGSC0003DMG400038229 | 1 | /source=BGI; /ID=PGSC0003DMG400038229; /name=Conserved gene of unknown function |
| PGSC0003DMG400046606 | 1 | /source=BGI; /ID=PGSC0003DMG400046606; /name=Gene of unknown function |
| PGSC0003DMG400036746 | 1 | /source=BGI; /ID=PGSC0003DMG400036746; /name=Polyprotein |
| PGSC0003DMG400008762 | 1 | /source=BGI; /ID=PGSC0003DMG400008762; /name=Class S F-box protein |
| PGSC0003DMG400043440 | 1 | /source=BGI; /ID=PGSC0003DMG400043440; /name=Gene of unknown function |
| PGSC0003DMG400041137 | 1 | /source=BGI; /ID=PGSC0003DMG400041137; /name=Gene of unknown function |
| PGSC0003DMG400040576 | 1 | /source=BGI; /ID=PGSC0003DMG400040576; /name=Gene of unknown function |
| PGSC0003DMG400039543 | 1 | /source=BGI; /ID=PGSC0003DMG400039543; /name=Gene of unknown function |
| PGSC0003DMG400043368 | 1 | /source=BGI; /ID=PGSC0003DMG400043368; /name=Gene of unknown function |
| PGSC0003DMG400009445 | 1 | /source=BGI; /ID=PGSC0003DMG400009445; /name=Class S F-box protein |
| PGSC0003DMG400038616 | 1 | /source=BGI; /ID=PGSC0003DMG400038616; /name=Gag-pol polyprotein |
| PGSC0003DMG400034485 | 1 | /source=BGI; /ID=PGSC0003DMG400034485; /name=Gene of unknown function |
| PGSC0003DMG400035725 | 1 | /source=BGI; /ID=PGSC0003DMG400035725; /name=Gene of unknown function |
| PGSC0003DMG400036139 | 1 | /source=BGI; /ID=PGSC0003DMG400036139; /name=Polyprotein protein |
| PGSC0003DMG400046131 | 1 | /source=BGI; /ID=PGSC0003DMG400046131; /name=Integrase core domain containing protein |
| PGSC0003DMG400046322 | 1 | /source=BGI; /ID=PGSC0003DMG400046322; /name=Gag-pro |
| PGSC0003DMG400037740 | 1 | /source=BGI; /ID=PGSC0003DMG400037740; /name=Gene of unknown function |
| PGSC0003DMG400042900 | 1 | /source=BGI; /ID=PGSC0003DMG400042900; /name=Integrase core domain containing protein |
| PGSC0003DMG400034937 | 1 | /source=BGI; /ID=PGSC0003DMG400034937; /name=Gene of unknown function |
| PGSC0003DMG400036858 | 1 | /source=BGI; /ID=PGSC0003DMG400036858; /name=Gene of unknown function |
| PGSC0003DMG400035838 | 1 | /source=BGI; /ID=PGSC0003DMG400035838; /name=Gene of unknown function |
| PGSC0003DMG400037640 | 1 | /source=BGI; /ID=PGSC0003DMG400037640; /name=Gene of unknown function |
| PGSC0003DMG400045285 | 1 | /source=BGI; /ID=PGSC0003DMG400045285; /name=Conserved gene of unknown function |
| PGSC0003DMG400047158 | 1 | /source=BGI; /ID=PGSC0003DMG400047158; /name=Retrotransposon gag protein |
| PGSC0003DMG400009446 | 1 | /source=BGI; /ID=PGSC0003DMG400009446; /name=Class S F-box protein |
| PGSC0003DMG400044077 | 1 | /source=BGI; /ID=PGSC0003DMG400044077; /name=Gene of unknown function |
| PGSC0003DMG400037318 | 1 | /source=BGI; /ID=PGSC0003DMG400037318; /name=Polyprotein |
| PGSC0003DMG400035427 | 1 | /source=BGI; /ID=PGSC0003DMG400035427; /name=Gag-pol polyprotein |
| PGSC0003DMG400043130 | 1 | /source=BGI; /ID=PGSC0003DMG400043130; /name=Conserved gene of unknown function |
| PGSC0003DMG400044893 | 1 | /source=BGI; /ID=PGSC0003DMG400044893; /name=Conserved gene of unknown function |
| PGSC0003DMG400034800 | 1 | /source=BGI; /ID=PGSC0003DMG400034800; /name=Gene of unknown function |
| PGSC0003DMG400027942 | 1 | /source=BGI; /ID=PGSC0003DMG400027942; /name=Gene of unknown function |
| PGSC0003DMG400038677 | 1 | /source=BGI; /ID=PGSC0003DMG400038677; /name=Conserved gene of unknown function |
| PGSC0003DMG400045939 | 1 | /source=BGI; /ID=PGSC0003DMG400045939; /name=Gag-pol polyprotein |
| PGSC0003DMG400041561 | 1 | /source=BGI; /ID=PGSC0003DMG400041561; /name=Gene of unknown function |
| PGSC0003DMG400042295 | 1 | /source=BGI; /ID=PGSC0003DMG400042295; /name=Gene of unknown function |
| PGSC0003DMG400046730 | 1 | /source=BGI; /ID=PGSC0003DMG400046730; /name=Transposon protein, CACTA, En/Spm sub-class |
| PGSC0003DMG400047308 | 1 | /source=BGI; /ID=PGSC0003DMG400047308; /name=Conserved gene of unknown function |
| PGSC0003DMG400039887 | 1 | /source=BGI; /ID=PGSC0003DMG400039887; /name=Integrase core domain containing protein |
| PGSC0003DMG400036864 | 1 | /source=BGI; /ID=PGSC0003DMG400036864; /name=Conserved gene of unknown function |
| PGSC0003DMG400044073 | 1 | /source=BGI; /ID=PGSC0003DMG400044073; /name='chromo' domain containing protein |
| PGSC0003DMG400036556 | 1 | /source=BGI; /ID=PGSC0003DMG400036556; /name=Gene of unknown function |
| PGSC0003DMG400041428 | 1 | /source=BGI; /ID=PGSC0003DMG400041428; /name=Gene of unknown function |
| PGSC0003DMG403009595 | 1 | /source=BGI; /ID=PGSC0003DMG403009595; /name=Glutamate-ammonia ligase |
| PGSC0003DMG402009595 | 1 | /source=BGI; /ID=PGSC0003DMG402009595; /name=Nodulin / glutamate-ammonia ligase |
| PGSC0003DMG401009595 | 1 | /source=BGI; /ID=PGSC0003DMG401009595; /name=Catalytic/ glutamate-ammonia ligase |
| PGSC0003DMG400040619 | 1 | /source=BGI; /ID=PGSC0003DMG400040619; /name=Cytochrome P450 |
| PGSC0003DMG400042490 | 9 | /source=BGI; /ID=PGSC0003DMG400042490; /name=Conserved gene of unknown function |
| PGSC0003DMG400035892 | 9 | /source=BGI; /ID=PGSC0003DMG400035892; /name='chromo' domain containing protein |
| PGSC0003DMG400022078 | 9 | /source=BGI; /ID=PGSC0003DMG400022078; /name=DNA damage-binding protein |
| PGSC0003DMG400022079 | 9 | /source=BGI; /ID=PGSC0003DMG400022079; /name=Conserved gene of unknown function |
| PGSC0003DMG400035739 | 9 | /source=BGI; /ID=PGSC0003DMG400035739; /name=GDSL-motif lipase/hydrolase family protein |
| PGSC0003DMG400036988 | 9 | /source=BGI; /ID=PGSC0003DMG400036988; /name=Gene of unknown function |
| PGSC0003DMG400023049 | 9 | /source=BGI; /ID=PGSC0003DMG400023049; /name=Conserved gene of unknown function |
| PGSC0003DMG400041840 | 9 | /source=BGI; /ID=PGSC0003DMG400041840; /name=Gene of unknown function |
| PGSC0003DMG400037086 | 9 | /source=BGI; /ID=PGSC0003DMG400037086; /name=Gene of unknown function |
| PGSC0003DMG400036701 | 9 | /source=BGI; /ID=PGSC0003DMG400036701; /name=Gene of unknown function |
| PGSC0003DMG400042269 | 9 | /source=BGI; /ID=PGSC0003DMG400042269; /name=Gene of unknown function |
| PGSC0003DMG400037177 | 9 | /source=BGI; /ID=PGSC0003DMG400037177; /name=Polyprotein protein |
| PGSC0003DMG400040218 | 9 | /source=BGI; /ID=PGSC0003DMG400040218; /name=Gene of unknown function |
| PGSC0003DMG400044927 | 9 | /source=BGI; /ID=PGSC0003DMG400044927; /name=Gene of unknown function |
| PGSC0003DMG400046399 | 9 | /source=BGI; /ID=PGSC0003DMG400046399; /name=Gene of unknown function |
| PGSC0003DMG400040777 | 9 | /source=BGI; /ID=PGSC0003DMG400040777; /name=Gene of unknown function |
| PGSC0003DMG400040847 | 9 | /source=BGI; /ID=PGSC0003DMG400040847; /name=Gene of unknown function |
| PGSC0003DMG400036232 | 9 | /source=BGI; /ID=PGSC0003DMG400036232; /name=Gene of unknown function |
| PGSC0003DMG400046264 | 9 | /source=BGI; /ID=PGSC0003DMG400046264; /name=Conserved gene of unknown function |
| PGSC0003DMG400042069 | 9 | /source=BGI; /ID=PGSC0003DMG400042069; /name=Gene of unknown function |
| PGSC0003DMG400041527 | 9 | /source=BGI; /ID=PGSC0003DMG400041527; /name=Gene of unknown function |
| PGSC0003DMG400042535 | 9 | /source=BGI; /ID=PGSC0003DMG400042535; /name=Gene of unknown function |
| PGSC0003DMG400042181 | 9 | /source=BGI; /ID=PGSC0003DMG400042181; /name=Gene of unknown function |
| PGSC0003DMG401017675 | 9 | /source=BGI; /ID=PGSC0003DMG401017675; /name=Binding protein |
| PGSC0003DMG402017675 | 9 | /source=BGI; /ID=PGSC0003DMG402017675; /name=Binding protein |
| PGSC0003DMG400017676 | 9 | /source=BGI; /ID=PGSC0003DMG400017676; /name=Gene of unknown function |
| PGSC0003DMG400035068 | 9 | /source=BGI; /ID=PGSC0003DMG400035068; /name=Gene of unknown function |
| PGSC0003DMG400041284 | 9 | /source=BGI; /ID=PGSC0003DMG400041284; /name=Polyprotein protein |
| PGSC0003DMG400038489 | 9 | /source=BGI; /ID=PGSC0003DMG400038489; /name=Gene of unknown function |
| PGSC0003DMG400035672 | 9 | /source=BGI; /ID=PGSC0003DMG400035672; /name=Gene of unknown function |
| PGSC0003DMG400034653 | 9 | /source=BGI; /ID=PGSC0003DMG400034653; /name=Gene of unknown function |
| PGSC0003DMG400045590 | 9 | /source=BGI; /ID=PGSC0003DMG400045590; /name=Conserved gene of unknown function |
| PGSC0003DMG400038212 | 9 | /source=BGI; /ID=PGSC0003DMG400038212; /name=Conserved gene of unknown function |
| PGSC0003DMG400041881 | 9 | /source=BGI; /ID=PGSC0003DMG400041881; /name=Conserved gene of unknown function |
| PGSC0003DMG400035526 | 9 | /source=BGI; /ID=PGSC0003DMG400035526; /name=Gene of unknown function |
| PGSC0003DMG400039776 | 9 | /source=BGI; /ID=PGSC0003DMG400039776; /name=Gene of unknown function |
| PGSC0003DMG400040983 | 9 | /source=BGI; /ID=PGSC0003DMG400040983; /name=Gene of unknown function |
| PGSC0003DMG400017677 | 9 | /source=BGI; /ID=PGSC0003DMG400017677; /name=Gene of unknown function |
| PGSC0003DMG400036037 | 9 | /source=BGI; /ID=PGSC0003DMG400036037; /name=Gene of unknown function |
| PGSC0003DMG400043790 | 9 | /source=BGI; /ID=PGSC0003DMG400043790; /name=Gene of unknown function |
| PGSC0003DMG400035811 | 9 | /source=BGI; /ID=PGSC0003DMG400035811; /name=Gene of unknown function |
| PGSC0003DMG400035202 | 9 | /source=BGI; /ID=PGSC0003DMG400035202; /name=Gene of unknown function |
| PGSC0003DMG400037808 | 9 | /source=BGI; /ID=PGSC0003DMG400037808; /name=Gene of unknown function |
| PGSC0003DMG400040333 | 9 | /source=BGI; /ID=PGSC0003DMG400040333; /name=Gene of unknown function |
| PGSC0003DMG400035640 | 9 | /source=BGI; /ID=PGSC0003DMG400035640; /name=Gene of unknown function |
| PGSC0003DMG400036120 | 9 | /source=BGI; /ID=PGSC0003DMG400036120; /name=Gene of unknown function |
| PGSC0003DMG400044259 | 9 | /source=BGI; /ID=PGSC0003DMG400044259; /name=Gene of unknown function |
| PGSC0003DMG400043763 | 9 | /source=BGI; /ID=PGSC0003DMG400043763; /name=Gene of unknown function |
| PGSC0003DMG400046263 | 9 | /source=BGI; /ID=PGSC0003DMG400046263; /name=Gene of unknown function |
| PGSC0003DMG400041675 | 9 | /source=BGI; /ID=PGSC0003DMG400041675; /name=Gene of unknown function |
| PGSC0003DMG400042353 | 9 | /source=BGI; /ID=PGSC0003DMG400042353; /name=Gene of unknown function |
| PGSC0003DMG400046662 | 9 | /source=BGI; /ID=PGSC0003DMG400046662; /name=Gene of unknown function |
| PGSC0003DMG400037435 | 9 | /source=BGI; /ID=PGSC0003DMG400037435; /name=Gene of unknown function |
| PGSC0003DMG400046074 | 9 | /source=BGI; /ID=PGSC0003DMG400046074; /name=Conserved gene of unknown function |
| PGSC0003DMG400042173 | 9 | /source=BGI; /ID=PGSC0003DMG400042173; /name=Conserved gene of unknown function |
| PGSC0003DMG400042624 | 9 | /source=BGI; /ID=PGSC0003DMG400042624; /name=Conserved gene of unknown function |
| PGSC0003DMG400039766 | 9 | /source=BGI; /ID=PGSC0003DMG400039766; /name=Gene of unknown function |
| PGSC0003DMG400031396 | 9 | /source=BGI; /ID=PGSC0003DMG400031396; /name=ATP synthase subunit d |
| PGSC0003DMG400037142 | 9 | /source=BGI; /ID=PGSC0003DMG400037142; /name=Integrase core domain containing protein |
| PGSC0003DMG400042084 | 9 | /source=BGI; /ID=PGSC0003DMG400042084; /name=Gene of unknown function |
| PGSC0003DMG400047202 | 9 | /source=BGI; /ID=PGSC0003DMG400047202; /name=Integrase core domain containing protein |
| PGSC0003DMG400043694 | 9 | /source=BGI; /ID=PGSC0003DMG400043694; /name=Gene of unknown function |
| PGSC0003DMG400041414 | 9 | /source=BGI; /ID=PGSC0003DMG400041414; /name=Gene of unknown function |
| PGSC0003DMG400046532 | 9 | /source=BGI; /ID=PGSC0003DMG400046532; /name=Gene of unknown function |
| PGSC0003DMG400040938 | 9 | /source=BGI; /ID=PGSC0003DMG400040938; /name=Gene of unknown function |
| PGSC0003DMG400002333 | 9 | /source=BGI; /ID=PGSC0003DMG400002333; /name=Desacetoxyvindoline 4-hydroxylase |
| PGSC0003DMG400040698 | 9 | /source=BGI; /ID=PGSC0003DMG400040698; /name=Gene of unknown function |
| PGSC0003DMG400035514 | 9 | /source=BGI; /ID=PGSC0003DMG400035514; /name=Integrase core domain containing protein |
| PGSC0003DMG400043463 | 9 | /source=BGI; /ID=PGSC0003DMG400043463; /name=Gene of unknown function |
| PGSC0003DMG400036140 | 9 | /source=BGI; /ID=PGSC0003DMG400036140; /name=Gene of unknown function |
| PGSC0003DMG400044339 | 9 | /source=BGI; /ID=PGSC0003DMG400044339; /name=Integrase core domain containing protein |
| PGSC0003DMG400039615 | 9 | /source=BGI; /ID=PGSC0003DMG400039615; /name=Gene of unknown function |
| PGSC0003DMG400034587 | 9 | /source=BGI; /ID=PGSC0003DMG400034587; /name=DNAJ heat shock N-terminal domain-containing protein |
| PGSC0003DMG400036182 | 9 | /source=BGI; /ID=PGSC0003DMG400036182; /name=Gene of unknown function |
| PGSC0003DMG400002318 | 9 | /source=BGI; /ID=PGSC0003DMG400002318; /name=DNA binding protein |
| PGSC0003DMG400041888 | 9 | /source=BGI; /ID=PGSC0003DMG400041888; /name=Gene of unknown function |
| PGSC0003DMG400035695 | 9 | /source=BGI; /ID=PGSC0003DMG400035695; /name=Conserved gene of unknown function |
| PGSC0003DMG400042944 | 9 | /source=BGI; /ID=PGSC0003DMG400042944; /name=Gene of unknown function |
| PGSC0003DMG400039133 | 9 | /source=BGI; /ID=PGSC0003DMG400039133; /name=Gene of unknown function |
| PGSC0003DMG400038059 | 9 | /source=BGI; /ID=PGSC0003DMG400038059; /name=Gene of unknown function |
| PGSC0003DMG400041001 | 9 | /source=BGI; /ID=PGSC0003DMG400041001; /name=Gene of unknown function |
| PGSC0003DMG400043248 | 9 | /source=BGI; /ID=PGSC0003DMG400043248; /name=Gene of unknown function |
| PGSC0003DMG400047073 | 9 | /source=BGI; /ID=PGSC0003DMG400047073; /name=Conserved gene of unknown function |
| PGSC0003DMG400034391 | 9 | /source=BGI; /ID=PGSC0003DMG400034391; /name=Integrase core domain containing protein |
| PGSC0003DMG400034618 | 9 | /source=BGI; /ID=PGSC0003DMG400034618; /name=Conserved gene of unknown function |
| PGSC0003DMG400039065 | 9 | /source=BGI; /ID=PGSC0003DMG400039065; /name=Ulp1 protease family, C-terminal catalytic domain containing protein |
| PGSC0003DMG400045721 | 9 | /source=BGI; /ID=PGSC0003DMG400045721; /name=Gene of unknown function |
| PGSC0003DMG400036370 | 9 | /source=BGI; /ID=PGSC0003DMG400036370; /name=Conserved gene of unknown function |
| PGSC0003DMG400044333 | 9 | /source=BGI; /ID=PGSC0003DMG400044333; /name=Gene of unknown function |
| PGSC0003DMG400021000 | 9 | /source=BGI; /ID=PGSC0003DMG400021000; /name=Homeodomain protein HOX3 |
| PGSC0003DMG400036168 | 9 | /source=BGI; /ID=PGSC0003DMG400036168; /name=Gene of unknown function |
| PGSC0003DMG400021001 | 9 | /source=BGI; /ID=PGSC0003DMG400021001; /name=Gene of unknown function |
| PGSC0003DMG400044208 | 9 | /source=BGI; /ID=PGSC0003DMG400044208; /name=Gene of unknown function |
| PGSC0003DMG400023102 | 9 | /source=BGI; /ID=PGSC0003DMG400023102; /name=Homeobox protein GLABRA2 |
| PGSC0003DMG400035810 | 9 | /source=BGI; /ID=PGSC0003DMG400035810; /name=Conserved gene of unknown function |
| PGSC0003DMG400023100 | 9 | /source=BGI; /ID=PGSC0003DMG400023100; /name=CDPK adapter protein 1 |
| PGSC0003DMG400023099 | 9 | /source=BGI; /ID=PGSC0003DMG400023099; /name=WRKY transcription factor |
| PGSC0003DMG401023104 | 9 | /source=BGI; /ID=PGSC0003DMG401023104; /name=Conserved gene of unknown function |
| PGSC0003DMG400023105 | 9 | /source=BGI; /ID=PGSC0003DMG400023105; /name=Gene of unknown function |
| PGSC0003DMG403023104 | 9 | /source=BGI; /ID=PGSC0003DMG403023104; /name=Gene of unknown function |
| PGSC0003DMG402023104 | 9 | /source=BGI; /ID=PGSC0003DMG402023104; /name=Gene of unknown function |
| PGSC0003DMG400023098 | 9 | /source=BGI; /ID=PGSC0003DMG400023098; /name=GPI-anchor transamidase |
| PGSC0003DMG400023103 | 9 | /source=BGI; /ID=PGSC0003DMG400023103; /name=SUMO protein |
| PGSC0003DMG400042660 | 9 | /source=BGI; /ID=PGSC0003DMG400042660; /name=Gene of unknown function |
| PGSC0003DMG400042192 | 9 | /source=BGI; /ID=PGSC0003DMG400042192; /name=Conserved gene of unknown function |
