# Supplementary material for: Cytosolic glutamine synthetase is important for photosynthetic efficiency and water use efficiency in potato as revealed by high-throughput sequencing QTL analysis
Source: Theor Appl Genet. 2015 Jul 12;128(11):2143–53. doi: 10.1007/s00122-015-2573-2 (PMC4624824; doi:10.1007/s00122-015-2573-2)
Supplement: Supplementary file 4 — Supplementary material 4 (DOCX 15 kb) [file 122_2015_2573_MOESM4_ESM.docx]

|  | AA | Full length? |  |  |  | Expression (FPKM) - leaf | |
| --- | --- | --- | --- | --- | --- | --- | --- |
| Gene Name |  |  | Gene ID | Transcript ID | Peptide ID | DM1-3 516 R44 | RH89-039-16 |
| Glutamine synthetase | 446 | Yes | PGSC0003DMG400004355 | PGSC0003DMT400011133 | PGSC0003DMP400007746 | 698.9 | 1101.4 |
| Glutamine synthetase | 356 | Yes | PGSC0003DMG400023620 | PGSC0003DMT400060733 | PGSC0003DMP400040866 | 20.62 | 31.95 |
| Glutamine synthetase | 356 | Yes | PGSC0003DMG400014592 | PGSC0003DMT400037822 | PGSC0003DMP400025701 | 0.64 | 1.51 |
| Glutamine synthetase | 210 | No | PGSC0003DMG400017703 | PGSC0003DMT400045640 | PGSC0003DMP400030933 | 42.8 | 89.19 |
| Glutamine synthetase | 254 | No | PGSC0003DMG400013235 | PGSC0003DMT400034421 | PGSC0003DMP400023400 | 105.43 | 218.02 |
| Glutamine synthetase | 320 | Yes | PGSC0003DMG400014454 | PGSC0003DMT400037457 | PGSC0003DMP400025464 | 0 | 0 |
| Glutamine synthetase | 87 | No | PGSC0003DMG400014115 | PGSC0003DMT400036605 | PGSC0003DMP400024818 | 342.65 | 399.09 |
| Catalytic/ glutamate-ammonia ligase | 301 | Yes | PGSC0003DMG401009595 | PGSC0003DMT400024814 | PGSC0003DMP400016952 | 2.12 | 1.48 |
| Glutamate-ammonia ligase | 320 | Yes | PGSC0003DMG403009595 | PGSC0003DMT400024816 | PGSC0003DMP400016954 | 5.19 | 2.29 |
| Nodulin / glutamate-ammonia ligase | 141 | No | PGSC0003DMG402009595 | PGSC0003DMT400024815 | PGSC0003DMP400016953 | 4.72 | 0 |
| Glutamate-ammonia ligase | 469 | Yes | PGSC0003DMG400028171 | PGSC0003DMT400072398 | PGSC0003DMP400048953 | 0 | 0 |
| Glutamate-ammonia ligase | 850 | Too long | PGSC0003DMG400028277 | PGSC0003DMT400072666 | PGSC0003DMP400049162 | 0 | 0 |
| Glutamate-ammonia ligase | 350 | Yes | PGSC0003DMG400028276 | PGSC0003DMT400072665 | PGSC0003DMP400049161 | 3.93 | 3.15 |
| Glutamate-ammonia ligase | 173 | No | PGSC0003DMG401019012 | PGSC0003DMT400048929 | PGSC0003DMP400033075 | 0.73 | 0.55 |
| Glutamate-ammonia ligase | 276 | Yes | PGSC0003DMG400019014 | PGSC0003DMT400048935 | PGSC0003DMP400033078 | 0.98 | 1.71 |
| Glutamate-ammonia ligase | 176 | No | PGSC0003DMG400019013 | PGSC0003DMT400048932 | PGSC0003DMP400033077 | 0.71 | 0.53 |
| Nodulin / glutamate-ammonia ligase | 154 | No | PGSC0003DMG400019011 | PGSC0003DMT400048927 | PGSC0003DMP400033074 | 0 | 0.33 |
